# Elucidation of Vasodilation Response and Structure Activity Relationships of *N*^2^,*N*^4^*-*Disubstituted Quinazoline 2,4-Diamines in a Rat Pulmonary Artery Model

**DOI:** 10.3390/molecules24020281

**Published:** 2019-01-14

**Authors:** Tamkeen Urooj Paracha, Nattakarn Pobsuk, Nattapas Salaloy, Praphasri Suphakun, Dumrongsak Pekthong, Supa Hannongbua, Kiattawee Choowongkomon, Nantaka Khorana, Prapapan Temkitthawon, Kornkanok Ingkaninan, M. Paul Gleeson, Krongkarn Chootip

**Affiliations:** 1 Department of Pharmacy Practice, Faculty of Pharmaceutical Sciences, Naresuan University, Phitsanulok 65000, Thailand; ctamz@hotmail.com (T.U.P.); dumrongsakp@yahoo.com (D.P.); 2 Department of Chemistry, Faculty of Science, Kasetsart University, Bangkok 10900, Thailand; sai-nurse@hotmail.com (N.P.); arthahaha@hotmail.com (N.S.); fscisph@ku.ac.th (S.H.); 3 Department of Biochemistry, Faculty of Science, Kasetsart University, Bangkok 10900, Thailand; cup14t@gmail.com (P.S.); fsciktc@ku.ac.th (K.C.); 4 Division of Pharmaceutical Sciences, School of Pharmaceutical Sciences, University of Phayao, Phayao 56000, Thailand; nantaka@hotmail.com; 5 Department of Pharmaceutical Chemistry and Pharmacognosy, Faculty of Pharmaceutical Sciences and Center of Excellence for Innovation in Chemistry, Naresuan University, Phitsanulok 65000, Thailand; prapapantem@gmail.com (P.T.); kornkanoki@nu.ac.th (K.I.); 6 Department of Biomedical Engineering, Faculty of Engineering, King Mongkut’s Institute of Technology, Ladkrabang, Bangkok 10520, Thailand; 7 Department of Physiology, Faculty of Medical Science, Naresuan University, Phitsanulok 65000, Thailand

**Keywords:** vasodilation, rat pulmonary artery, PDE-5 inhibitor, *N*^2^,*N*^4^-diamino quinazoline, cheminformatics

## Abstract

Pulmonary arterial hypertension (PAH) is a rare and progressive disease arising from various etiologies and pathogenesis. PAH decreases life expectancy due to pulmonary vascular remodeling, elevation of mean pulmonary arterial pressure, and ultimately progresses to heart failure. While clinical treatments are available to reduce the associated symptoms, a complete cure has yet to be found. Phosphodiesterase-5 (PDE-5) inhibition has been identified as a possible intervention point in PAH treatment. The functional vasodilation response to *N*^2^,*N*^4^-diamino quinazoline analogues with differing PDE-5 inhibitory activities and varying physicochemical properties were assessed in both endothelium-intact and denuded rat pulmonary arteries to gain greater insight into their mode of action. All analogues produced vasorelaxant effects with EC50s ranging from 0.58 ± 0.22 µM to ˃30 µM. It was observed that vasodilation response in intact vessels was highly correlated with that of denuded vessels. The ~10% drop in activity is consistent with a loss of the nitric oxide mediated cyclic guanosine monophosphate (NO/cGMP) pathway in the latter case. A moderate correlation between the vasodilation response and PDE-5 inhibitory activity in the intact vessels was observed. Experimental protocol using the alpha-adrenergic (α_1_) receptor agonist, phenylephrine (PE), was undertaken to assess whether quinazoline derivatives showed competitive behavior similar to the α_1_ receptor blocker, prazosin, itself a quinazoline derivative, or to the PDE-5 inhibitor, sildenafil. Competitive experiments with the α_1_-adrenergic receptor agonist point to quinazoline derivatives under investigation here act via PDE-5 inhibition and not the former. The pre-incubation of pulmonary arterial rings with quinazoline test compounds (10 μM) reduced the contractile response to PE around 40–60%. The most promising compound (9) possessed ~32 folds higher selectivity in terms of vasodilation to its mammalian A549 cell cytotoxicity. This study provides experi0 0mental basis for PDE-5 inhibition as the mode of action for vasodilation by *N*^2^,*N*^4^-diamino quinazoline analogues along with their safety studies that may be beneficial in the treatment of various cardiovascular pathologies.

## 1. Introduction

Pulmonary arterial hypertension (PAH) is a rare (~15 cases/million) [1] and progressive disease based on diverse etiologies [2]. This disease reduces life expectancy due to pulmonary vascular remodeling, elevation of mean pulmonary arterial pressure, right ventricular (RV) hypertrophy, and heart failure [3]. The causative factors responsible for progression of PAH include endothelial dysfunction, resulting in an imbalance of vasoconstrictors and vasodilators. The former leads to more extensive vascular smooth muscle remodeling and increases pulmonary arterial pressure and advancement of PAH [4,5]. A complete cure of this fatal disease is not yet available, however, a number of treatment options can be used to deal with the clinical symptoms and to increase survival rates [6]. Treatments for PAH exists as monotherapy and combination therapies [7]. These include supplemental oxygen, anticoagulants, and diuretics [8]. Only 7–8% patients showing a positive vasoreactivity test upon right heart catheterization respond to calcium channel blockers (CCBs) with sustained cardiac output [8,9]. Treatment with prostacyclin analogues (intravenous and subcutaneous) have also been approved for PAH [9]. Endothelin receptor antagonists (Bosentan, etc.) are being used as first line oral treatment [10], however, their shortcomings include significant expense, catheter induced sepsis, relatively low response rates, and risk of drug interactions.

Phosphodiesterase-5 (PDE-5) inhibitors are considered to improve clinical hemodynamics in combination with other treatment regimens based on patient response and tolerability studies [11,12]. This was prompted by recognizing the upregulation of PDE-5 in pulmonary hypoxic conditions and PAH [13,14]. PDE-5 inhibitors, therefore, offer great potential as therapeutic agents for PAH. PDE-5 enzyme is abundantly expressed in smooth muscle cells of the penis, lungs, kidney, spleen, as well as platelets, all of which rely heavily on the cyclic guanosine monophosphate (cGMP) signaling cascade [15,16]. Marketed inhibitors of the enzyme, such as sildenafil and vardenafil, are capable of producing acute vasodilatory effects through inhibition of cGMP hydrolysis. The cGMP accumulation augments the nitric oxide (NO) induced vascular smooth muscle relaxation [9,17]. Nevertheless, concerns associated with use of these inhibitors include dose-dependent adverse effects, including cross reactivity with other PDE isoforms (such as PDE-6 and PDE-1), leading to headaches, cutaneous flushing, nausea, and visual disturbances [18,19,20].

**Figure 1:**
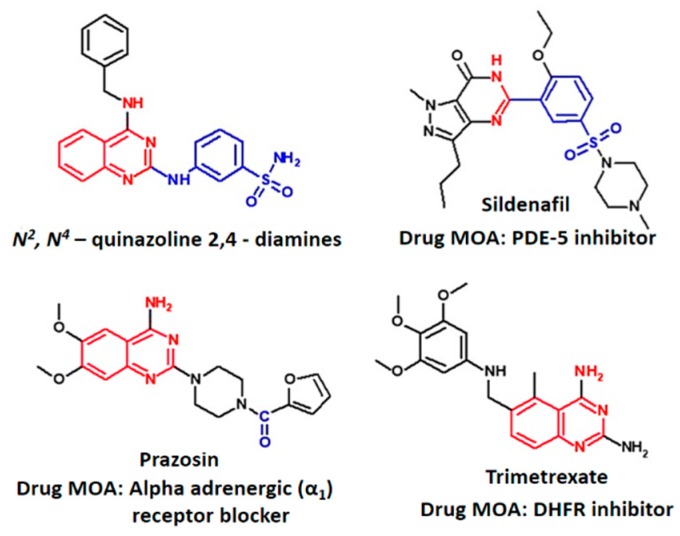
*N*^2^,*N*^4^*-*quinazoline 2,4-diamines vasodilator (compound **5**) shares structural similarity with known vasodilators, Prazosin (via the 2,4 diamino quinazoline) and Sildenafil (via Phosphodiesterase-5; PDE-5 inhibitory activity).

Quinazoline containing compounds display a variety of pharmacological effects, including antifungal [21], antimalarial [22], and antihypertensive effects (Figure 1). Vasodilation properties have been observed [23], mediated via modulation of the alpha 1-adrenergic (α_1_) receptor [24] and PDE-5 enzyme [25]. We recently reported the preparation and evaluation of *N*^2^,*N*^4^*-*diamino quinazoline analogues as inhibitors of the latter target [26]. The most active compound identified in this study, displayed as PDE-5 IC_50_ 0.072 µM, exhibited approximately 5-fold selectivity over PDE-6 inhibition and demonstrated ex-vivo vasodilator activity in a rat pulmonary artery model. Molecular modelling was used to confirm the binding mode of compounds to the enzyme and to prepare additional analogues for exploring enzyme structure activity relationships (SAR).

The purpose of this work was to profile in greater detail *N*^2^,*N*^4^*-*diamino quinazoline analogues (Table 1) in an ex-vivo vasodilation of a rat pulmonary artery model and compare them to other clinically marketed vasodilators. We have assessed the vasodilation response of a range of compounds with differing PDE-5 activities and varying physicochemical properties in both endothelium-intact and denuded pulmonary arteries. Endothelium-intact vessels represent normal and healthy arterial vessels having endothelial lining over smooth muscle cells, while endothelium-denuded vessels lack NO, prostacyclin (PGI_2_), endothelium derived hyperpolarizing factor (EDHF), and hydrogen sulfide (H_2_S) mediated pathways, due to the removal of the endothelial layer, and represent an improvised model of smooth muscle cells. Additionally, we compared and contrasted the phenotypic response of test compounds against the PDE-5 inhibitor, sildenafil, and the alpha-adrenergic (α_1_) receptor blocker, prazosin, as a means to better understand their mode of action. Since, *N*^2^,*N*^4^*-*diamino quinazoline analogues are confirmed PDE-5 inhibitors [26], they are structurally similar to α_1_ blocker, prazosin, suggesting the latter mechanism may also contribute to their phenotypic response. To this end, the compounds have also been assessed in terms of their competitive performance against the α_1_-adrenergic receptor agonist phenylephrine (PE).

## 2. Results

### 2.1. Vasorelaxant Effects of N^2^,N^4^-Diamino Quinazoline Analogues

**Figure 2:**
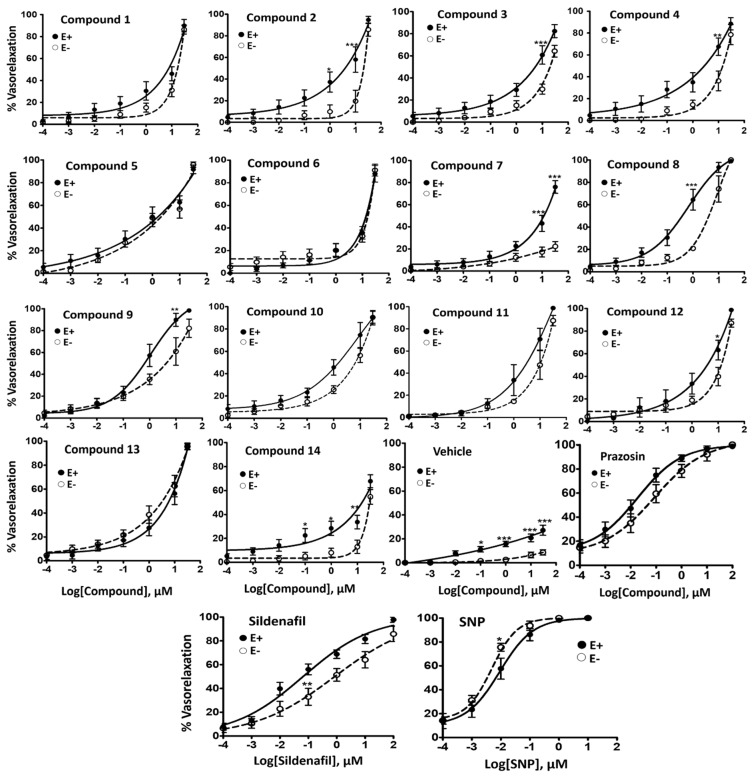
Concentration-relaxation curves for compounds **1** to **14**, vehicle (Dimethyl sulfoxide; DMSO), Prazosin, Sildenafil, Sodium nitroprusside (SNP) on PE pre-contracted endothelium-intact (E+), and endothelium-denuded (E−) pulmonary arterial (PA) vessels from rats. Relaxation to vehicle; DMSO at the same concentration as dissolved with test compounds in each concentration (maximum final concentration at 0.38%). All data were expressed as mean ± standard error of the mean (SEM) (n = 6). * *p* < 0.05, ** *p* < 0.01, *** *p* < 0.001 E+ vs. E−.

**Figure 3:**
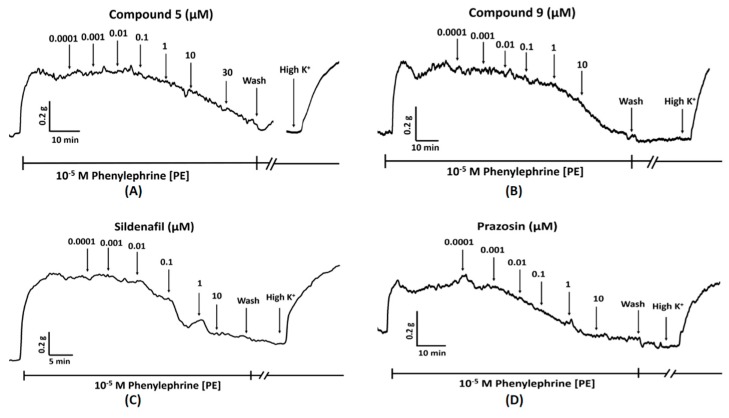
An example time course showing concentration-dependent relaxation of 10^−5^ M PE pre-contracted endothelium-intact (E+) pulmonary arterial rings, caused by cumulative addition of (**A**) compound **5**, (**B**) compound **9**, (**C**) Sildenafil, and (**D**) Prazosin each followed by the addition of high potassium solution (80 mM) to prove vessel viability. Scale bar within each trace represents real time on the x-axis while the y-axis symbolizes the vessel’s tension in the organ bath.

All compounds induced concentration-dependent relaxation in both endothelium-intact (EC_50_ ranging from 0.58–9.42 µM) and denuded pulmonary arterial rings (EC_50_ ranging from 1.15 to ˃30 µM) pre-contracted with PE (10^−5^ M) (Table 2 and Figure 2). Compound **8** was found most potent in endothelium-intact vessels (EC_50_ = 0.58 ± 0.22 µM; E_max_ = 98.80 ± 0.79%) while compound **5** displayed highest potency in endothelium-denuded vessels (EC_50_ = 1.15 ± 0.18 µM; E_max_ = 95.83 ± 2.40%). The vasorelaxant effect was observed to increase with a cumulative increase in the concentration of the compound in the organ bath with the passage of time. Representative tracing of the vasorelaxant effect of compounds **5** and **9** added cumulatively with the passage of real time in the organ bath is shown in Figure 3. Vasorelaxant effects were generally higher in endothelium-intact vessels compared to endothelium-denuded vessels at a given concentration. It reflected the increased potency of the test compounds in the presence of endothelium. Nevertheless, at higher concentrations, all compounds produced almost 100% vasorelaxation in endothelium-intact and denuded vessels except compounds **7** and **14**. No significant vasodilatory response in pulmonary arterial rings was observed for the negative control (DMSO).

**Figure 4:**
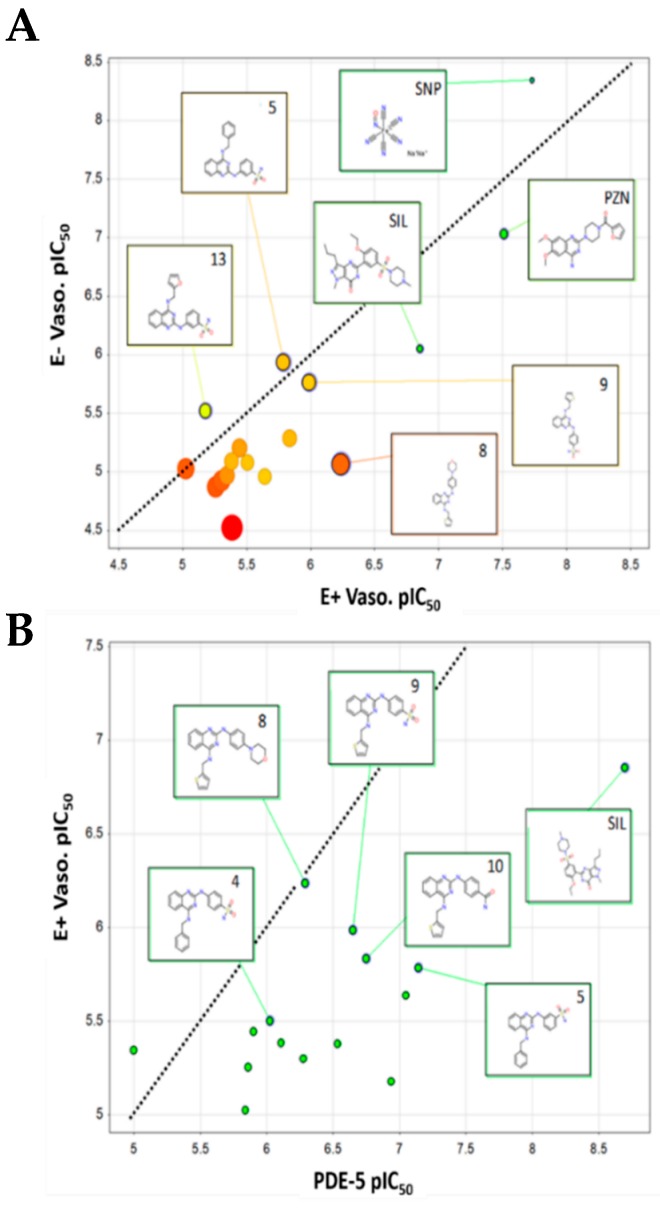
Relationship between the (**A**) vasodilation response in endothelium-denuded and endothelium-intact rat pulmonary arterial rings, (**B**) vasodilation response in endothelium-intact rat pulmonary arterial rings vs. in-vitro PDE-5 inhibitory activity.

Compounds **8**, **9**, **10**, and **5** were found to be most active in endothelium-intact vessels with an EC_50_ of 0.58, 1.03, 1.46, and 1.63 μM, respectively. No definitive SAR could be discerned, in part due to the relatively narrow distribution of EC_50_ values for the set (2.62 μM) compared to standard errors in the mean (SEM) ~1.5 on average. Compound **7** was an outlier as it was moderately active in endothelium-intact vessels (maximal relaxation; E_max_ = 76.03 ± 5.73%), but weakly active in endothelium-denuded vessels (E_max_ = 22.31 ± 4.40%) at the highest tested concentration (30 µM). The relationship between the pEC_50_s determined in endothelium-intact and endothelium-denuded vessels shows a strong correlation (Figure 4A), suggesting a mode of action involving endothelium is not operating (R^2^ = 0.77, RMSE = 3.5 μM). Compound **14**, which is borderline PDE-5 inactive, and contains a pyrimidine core, showed the lowest maximal relaxation in endothelium-intact vessels (E_max_ = 67.80 ± 5.34%). This would apparently confirm the importance of this target for the relaxation response observed in endothelium-intact pulmonary arteries.

### 2.2. Inhibitory Effect on Phenylephrine—Induced Contractile Response

**Figure 5:**
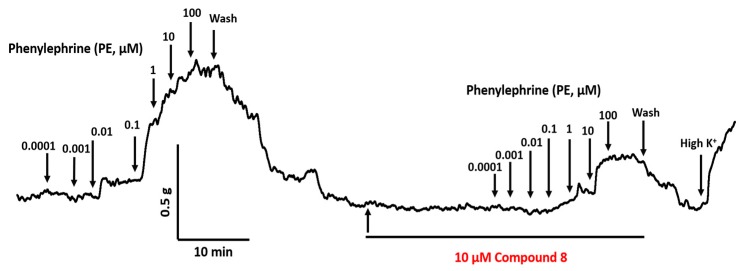
An example time course showing reduced contractions evoked by cumulative addition of PE (0.0001–100 µM) after 15 min incubation with 10 µM compound **8** followed by addition of high potassium solution (80 mM) to prove vessel viability.

Compounds **2**, **7**, **8**, **13,** prazosin, and sildenafil were further investigated in terms of their ability to inhibit PE-induced contraction. These experiments were undertaken to assess the pharmacological activity of quinazoline analogues against the structurally related α_1_ receptor blocker (prazosin). In contrast to previous vasorelaxant experiments, this protocol was developed to determine the inhibitory effect of pre-incubated (15 min) inhibitors (10 µM) on the concentration-dependent vasoconstriction induced by cumulative addition of PE (0.0001–100 µM) in endothelium-denuded pulmonary arterial rings. Figure 5 illustrates the real time contractile response shown by the pulmonary arterial ring upon cumulative addition of PE, which evoked a concentration-dependent vasoconstriction, increasing the vessel’s tension.

**Figure 6:**
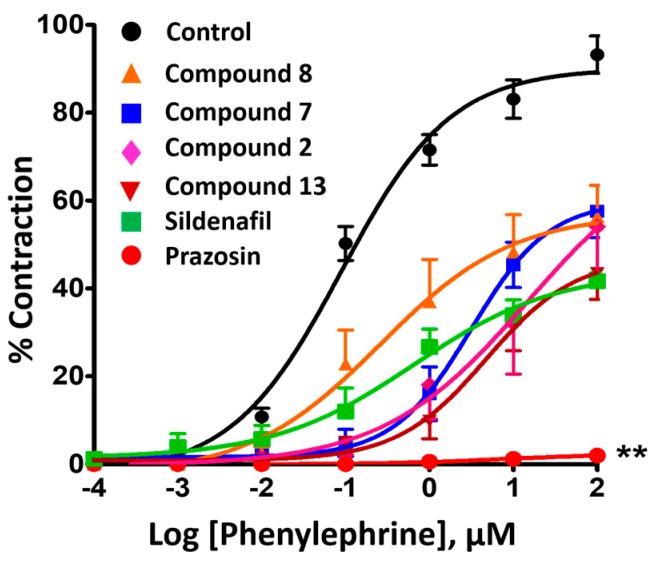
Concentration-response curves for PE-induced contraction (0.0001–100 µM) on endothelium-denuded (E−) pulmonary arterial rings after 15 min incubation of 10 µM test compounds. Contraction was presented as a percentage of the response in the presence compared to the absence of the tested compounds. All data were expressed as mean ± standard error of the mean (SEM) (n = 3–5). ** *p* < 0.01, comparing each curve vs. control (0.2% DMSO).

Figure 6 shows a plot of log[PE] concentration vs. pulmonary arterial contractile response induced by PE expressed as a percentage of the contraction in the presence and absence of tested compounds. The results revealed that compounds **2**, **7**, **8**, and **13** attenuated the maximal contraction induced by PE to 43.38 ± 5.90%, 57.57 ± 5.99%, 56.38 ± 7.10%, and 54.07 ± 11.69%, respectively. Prazosin, being a direct competitive blocker of the α_1_ agonist PE, inhibited the maximal contraction to 98.01 ± 1.02%. Sildenafil inhibited PE-provoked contraction similar to that of the test compounds (58.33 ± 1.77%). The control group (DMSO) displayed no significant effect (Figure 6).

### 2.3. Cytotoxicity and Solubility

An understanding of the selectivity window between the ex-vivo vasodilation response and general mammalian cytotoxicity is desirable for compound prioritization decisions. The cytotoxic effects of test compounds were therefore evaluated using an MTT assay in a mammalian A549 cell line. Test compounds exhibited cytotoxicity within the range of 2–30 µM. Compound **7** exhibited the highest cytotoxicity (IC_50_ = 2.91 ± 0.73 µM), while compound **9** exhibited the lowest cytotoxicity (IC_50_ = 32.67 ± 3.61 µM). Compounds **8** and **9**, the most potent vasodilators, showed 19.6 and 31.7 folds selectivity in terms of their vasodilation EC_50_ compared to their cytotoxic EC_50_. This compared to 22.3 and 148.5 folds selectivity in terms of their inhibitory PDE-5 EC_50_ compared to their cytotoxic EC_50._

The solubility of the compounds under investigation here were assessed in phosphate buffer at pH 7.4 using the equilibrium shake-flask method [27]. The quinazoline analogues displayed solubility ranging from 0.01 to 1.02 mg/mL. Sildenafil displayed a considerably larger solubility of 20.83 mg/mL. In comparison, the solubility of prazosin was determined to be 0.55 mg/mL, more akin to the quinazoline derivatives under investigation here.

## 3. Discussion

Vasorelaxation is the vascular response regulated by both endothelium dependent and independent signaling pathways. Our organ bath experiments demonstrated evidence for concentration-dependent vasorelaxant effects of quinazoline derivatives (0.0001–30 µM) in endothelium-intact vessels. All test compounds were found to increase the concentration-dependent %vasorelaxation upon cumulative addition in the organ bath. Compound **8** produced a 50% vasorelaxation response at the lowest concentration observed, corresponding to 0.58 ± 0.22 µM. This was 4-fold greater than sildenafil (0.14 ± 0.05 µM) and ~19 times higher than prazosin (0.031 ± 0.01 µM). DMSO itself possesses the capacity to relax vessels by increasing cGMP and decreasing Ca^2+^ sensitivity, therefore, %DMSO was controlled [28]. The highest concentration of the test compounds (30 µM) contained a final DMSO volume of 0.38% in 10 mL organ bath, therefore, further higher concentrations were not tested as most of the compounds reached 100% maximal relaxation before 30 µM.

All test compounds were capable of producing concentration-dependent vasorelaxation even in the vessel whose endothelium was mechanically removed, except compound **7**. This proved to be a favourable response of the compounds, as progressive PAH is associated to endothelial dysfunction in pulmonary arteries. Hence, compounds possess the ability to produce an effective vasodilatory response regardless of endothelial dysfunction. Despite the reduced potency of the compounds in comparison with endothelium-intact vessels, major compounds did not display a significant difference, with the exception of compounds **1**, **4**, **8**, and **11** that were significantly less potent in endothelium-denuded vessels. The right shift of the concentration response curve in endothelium-denuded vessels is consistent with the loss of endothelial derived vasorelaxant pathways (NOS, PGI_2_, EDHF, and H_2_S) aiding the vasorelaxant ability of the compounds (i.e., production of cGMP for PDE-5 inhibitors). Compounds **5** and **9** were found to possess similar potency in both endothelium-denuded and endothelium-intact vessels.

The ability of compounds to inhibit PE-induced contraction was investigated in endothelium-denuded vessels as a means to exclude endothelium dependent vasodilation pathways. The %inhibition of PE-induced contraction varies with the concentration of compounds tested and the time of incubation. To minimize such variation, a single concentration (10 µM) of test compounds was chosen to study the inhibitory effects with a 15 min interval of the incubation period. Inhibition of PE-induced contraction between 40–60% was observed for quinazoline test compounds at 10 µM, similar to that attained by the PDE-5 inhibitor (sildenafil). This result was demonstrably different to the α_1_ receptor antagonist, prazosin, which is known to compete directly with PE to reduce vasoconstriction. Nevertheless, complete abolishment of the PE-induced contractile response by prazosin also could be related to its ~10 fold lower EC_50_ as compared to sildenafil and quinazoline compounds. 

Evidence suggesting that PDE-5 is the target of the quinazoline-based compounds under investigation here can be seen from the correlation of PDE-5 inhibitory activity (pIC_50_) against the vasodilation response (pEC_50_) in endothelium-denuded vessels (Figure 4B). A reasonably strong correlation exists between the two parameters (R^2^ = 0.51) that would further indicate that PDE-5 inhibitory activity is the key component of the ex-vivo vasorelaxant response of quinazoline analogues. Other factors, such as solubility and cell permeability, amongst other factors, could certainly account for the unexplained variance between in vitro and ex vivo activity data. Substructure searches of the ChEMBL database [29] using the *N*^2^,*N*^4^-diamino quinazoline scaffold identified a number of possible targets of these compounds (Tables S1 and S2 . These obviously include PDE-5 inhibitors and alpha-adrenergic (α_1_) receptor blockers, but also targets other potential links to vasodilation pathways, albeit invalidated. Dihydrofolate reductase (DHFR) is not a therapeutic intervention point for PAH treatments; rather, it is reported to affect the balance between NO and superoxide production in endothelial cells via endothelial nitric oxide synthase (eNOS) coupling [30], the latter of which is implicated in endothelium-dependent vasodilation [31]. Furthermore, analysis of DHFR chemotypes reveals that 2- and 4-amino groups remain unsubstituted, these being the key points of contact with the target enzyme. This rules out the quinazoline derivatives reported here as they are unable to bind to DHFR in this way. Other targets identified include the glucose transporter [32] and aldehyde dehydrogenase [33], however, the very low potency at these targets (high μM level) would also appear to rule these out.

**Figure 7:**
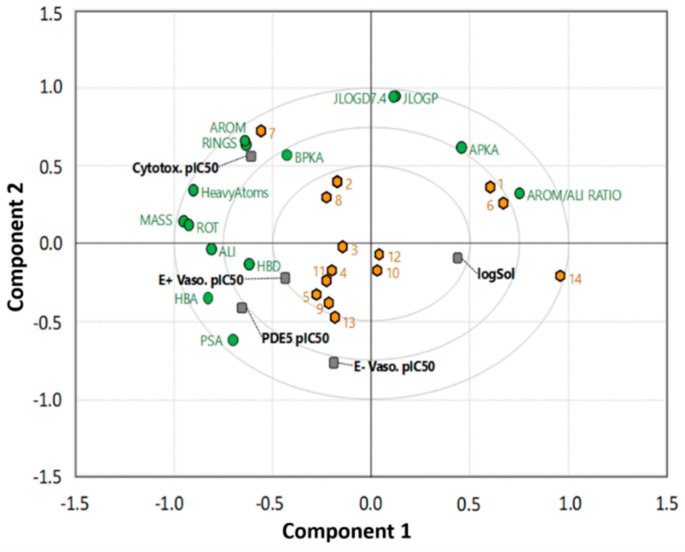
Relationship between the experimental PDE-5 inhibition, vasodilation, cytotoxicity, solubility, and other physicochemical properties as determined using principal components analysis (PCA).

A principal component analysis (PCA) model (Figure 7) was generated to better understand the relationship between the experimental biological activities and physicochemical parameters. The PCA was generated for all 14 quinazoline derivatives using 21 descriptors (five experimental, 16 computed) (Table S3). The PCA model fitted 14 compounds, explaining a total of 88% of the total variance in the dataset (Table S4). Components one and two are illustrated in the form of a loadings bi-plot in Figure 7, with each component corresponding to 42% and 7% of the variance, respectively. The results indicate that PDE-5 inhibitory activity correlates most strongly with the vasodilation response in endothelium-intact vessels as indicated by their clustering together in the lower left hand quadrant. In terms of physical properties, molecules with a greater number of donors (HBD) and acceptors (HBA) have greater PDE-5/vasodilation activities. LogP does not appear to play a significant role in defining the latter activities. It was found that the solubility of the compounds is inversely correlated with the vasodilation response (found on the opposite sides of component 1), indicating that optimization of both parameters in unison represents a challenge. Additionally, cytotoxicity is also found to be correlated to some extent with vasodilation.

## 4. Materials and Methods

### 4.1. Sample Preparation

Quinazoline analogues (*N*^2^,*N*^4^-quinazoline 2,4-diamines) were synthetized and purified to >95% as reported elsewhere [26]. All samples were dissolved in 100% DMSO and diluted with water to obtain the final test concentrations.

#### 4.1.1. Animals

Male Wistar rats (200–300 g) were acquired from Nomura Siam International Co, Ltd., Bangkok, Thailand. Standard environmental conditions of temperature (22 ± 1 °C), with an alternate 12 h light and dark cycle was maintained for animals. Free access to food and water was provided to the animals in the Center for Animal Research, Naresuan University (NUCAR), Phitsanlouk, Thailand. This study was approved and conducted in accordance to the guidelines from Naresuan University Animal Care and Use Committee (NUACUC; Animal Ethics Approval Number: NU-AE601021).

#### 4.1.2. Compounds and Solutions

Phenylephrine HCl, Acetylcholine chloride, Prazosin HCl, and Sodium nitroprusside dihydrate were all obtained from Sigma Chemical Company (St. Louis, MO, USA). Sildenafil was bought from the government pharmaceutical organization (GPO) of Thailand (Bangkok, Thailand). DMSO was acquired from VWR International Ltd. (Prolabo Chemicals, UK). All quinazoline analogues were dissolved in DMSO (100%) and diluted with distilled water.

### 4.2. Experimental Protocols

#### 4.2.1. Vascular Reactivity

After anesthesia with thiopental sodium (100 mg/kg, i.p.) (Anesthal^®^, Jagsonpsal Pharmaceutical Ltd., Haryana, India), rats were euthanized by exsanguination. Lungs were carefully excised, cleaned by the removal of connective tissue, and placed in cold physiological Krebs’ solution composed of (mM): NaCl 122; KCl 5; *N*-[2-hydroxyethyl] piperazine-*N*′-[2-ethane-sulfonic acid] (HEPES) 10; KH_2_PO_4_ 0.5; NaH_2_PO_4_ 0.5; MgCl_2_ 1; CaCl_2_ 1.8; and glucose 11 adjusted to pH 7.3 using 1 M NaOH. The pulmonary artery was isolated from the lungs and cut into rings approximately 2 mm in length before mounting in the organ bath chambers. Endothelial cells were mechanically removed in some experiments by gentle rubbing of the inner surface with forceps. Pulmonary arterial rings were mounted on intraluminal wires via stainless steel hooks maintaining the resting tension at 1 g and rings were equilibrated for 30 min before obtaining a stable contraction with 10^−5^ M phenylephrine (PE) [34,35]. The rings were immersed in 10 mL baths containing physiological Krebs’ solution at 37 °C, which was continuously bubbled with air. Force transducers were connected with intraluminal wires to measure isometric tension via a Mac Lab A/D converter (Chart V5, A.D. Instruments, Castle Hill, NSW, Australia). The functional integrity of the endothelium in the vessels was confirmed by vasorelaxation of ≥70% with sequential addition of 10^−5^ M acetylcholine (ACh) to pre-contracted vessels with 10^−5^ M PE. Relaxations ≤30% to 10^−5^ M ACh in vessels rubbed mechanically were considered successfully endothelium denuded.

Values are given as mean ± standard error of mean (S.E.M.) of n number of animals. The EC_50_ values (defined as the concentration of the test compound that induced 50% of the maximal relaxation) and E_max_ values (values of maximum relaxation) were obtained by actual concentration-response curve fitting using GraphPad Prism software (version 5.0, San Diego, CA, USA). The statistical analysis was conducted using un-paired Student’s t-test or one-way analysis of variance (ANOVA) between two groups followed by the Dunnet’s test as appropriate and assessed by two-way-ANOVA followed by Bonferroni’s post hoc test for comparison between multiple groups (Graph Pad Prism 5.0). *p*-value of <0.05 were considered statistically significant.

#### 4.2.2. Vasodilator Effects of Various Quinazoline Analogues

The experiment protocol was aimed to investigate and compare the vasodilator effects of 14 quinazoline analogues. Pulmonary arterial rings were stimulated with 10^−5^ M PE after maintenance of equilibrium in the organ bath for 45 min, keeping the resting tension to 1 g. Once the contraction reached a stable plateau, the test compound was added cumulatively (0.0001–30 µM) to the rings with both endothelium-intact and endothelium-denuded. The %relaxation was calculated as the percentage of the contraction in response to 10^−5^ M PE. The effect of the solvent (dimethyl sulfoxide; DMSO 0.38%) and positive control (sildenafil; Sil, sodium nitroprusside; SNP and prazosin were also evaluated in the same conditions as for each test compound. 80 mM high potassium Krebs’ solution (High K^+^ solution) was added to investigate the vessel’s viability at the end of experiment [34].

#### 4.2.3. Determination of Inhibitory Role of Quinazoline Analogues on PE-Induced Contraction

The protocol included the assessment of the inhibitory vasoconstriction effect of quinazoline test compounds on endothelium-denuded arterial rings. Briefly, the arterial rings were treated with the α_1_-receptor blocker (prazosin) or test compound at a concentration of 10 µM for 15 min before construction of the second concentration response curve with cumulative addition of PE (0.0001–100 µM). The same conditions were used to test the effects of the solvent (DMSO 0.2%). The results were obtained in percentages by comparison of the maximum contraction without treatment with the contraction in the presence of the test compound [24,36,37].

#### 4.2.4. Cytotoxicity

A 3-[4,5-dimehyl-2-thiazolyl]-2,5-diphenyl-2*H*-tetrazoliumbromide (MTT) assay was utilized to assess the concentration-response curves for quinazoline analogues as previously reported [38]. A549 cells (ATCC CCL-185, human alveolar basal epithelial cell line) were used to account for the general cytotoxicity of compounds. Cells were cultured in DMEM medium containing fetal bovine serum (FBS; 10%), penicillin (10 U/mL), and streptomycin (100 U/mL). The temperature was maintained at 37 °C with 5% CO_2._ Cells were seeded for 16–18 h in DMEM and incubated for 72 h with the test compounds. After incubation, MTT (5 mg/mL in normal saline) was added with DMEM and incubated for 3 h. Careful decantation of the medium and addition of DMSO (50 µL) was done. Absorbance values were recorded at 570 nm after 5 min mechanical shaking of the microplate (Sunrise microplate reader, Tecan).

The activity was presented as the inhibitory concentration required to produce a 50% response (IC_50_). Prism 6.0 GraphPad Software Inc., (San Diego, CA, USA) was utilized for data fitting. 0.5% DMSO was used as the negative control.

#### 4.2.5. Solubility

An equilibrium shake-flask technique was used to determine the solubility of samples in PBS at pH 7.4. Approximately 1 mg of sample was placed in a 10 mL glass screw top vial and dissolved in 1 mL of 0.02 M PBS at pH 7.4. The solution was sonicated for 1 h, followed by shaking for 6 h (IKA HS 260). The saturated solution was then filtered using a nylon syringe filter. A standard solution of each sample was prepared by dissolving a weighed amount of the sample in 1 mL of DMSO. The concentration of sample dissolved in the PBS solution was determined by comparing the peak area of the PBS and DMSO standard using an Agilent 1100 series HPLC with a Sunshell C8 column (Chromanik Technologies Inc., Osaka, Japan). A 100 µL injection of the aqueous sample and 5 µL of DMSO solution (pre-diluted 100 fold using DMSO) were used [27].

#### 4.2.6. Cheminformatics Analysis

Substructure searches of the ChEMBL database [29] were undertaken to identify the most probable targets associated with the 2,4-diamino quinazoline scaffold (Tables S1 and S2). Targets identified were further analyzed to identify possible interactions with pathways associated to vasodilation using searches of the primary literature.

The relationship between PDE-5 inhibitory activity, vasodilation, cytotoxicity, solubility, and computed parameters was assessed using principle components analysis (PCA). All computed parameters were obtained from Chemaxon JCHEM (San Diego, CA, USA) [39]. PCA models were built using all 14 quinazoline analogues and 19 descriptors in SIMCA P14 [40] (Table S3). Four cross-validated components were optimally fitted, explaining 88% of the total variance in the dataset. The first two components describe 70% of the variance and are therefore the focus of the discussion here.

## 5. Conclusions

*N*^2^,*N*^4^-disubstituted quinazoline 2,4-diamines produced vasorelaxation, acting through both endothelium and smooth muscle cells via inhibition of PDE-5. Despite structural similarity to the α_1_-adrenergic receptor blocker, prazosin, competitive experiments against PE in the rat pulmonary artery model appear to suggest they may function via a different mode of action. The quinazoline compounds displayed a phenotypic response in-line with the PDE-5 inhibitor, sildenafil, which is consistent with their known biochemical activities [26]. This was also consistent with the observations that quinazoline derivatives with greater PDE-5 inhibitory activity generally show improved activity in the vasodilation model.

Compounds **8** and **9** were identified as the most potent vasodilators, with EC_50_s of 0.58 and 1.03 μM, respectively. This corresponds to a 19.6 and 31.7 folds selectivity over their mammalian cytotoxicity. In comparison, sildenafil showed a vasodilation EC_50_ of 0.14 μM while prazosin, being a direct competitive blocker of the alpha-adrenergic (α_1_) receptor, showed an EC_50_ of 0.03 μM.

These results indicate that *N*^2^,*N*^4^-disubstituted quinazoline 2,4-diamines, as exemplified by compounds **8** and **9**, have clear potential as lead compounds for the treatment of vascular pathologies, including PAH. Additional work is needed to establish and improve the PDE-5 inhibitory and vasodilation activity, as well as increased compound solubility along with exploration of the pharmacokinetic profile of the series.

## Figures and Tables

**Table 1:**
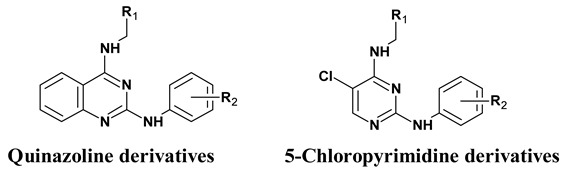
The structure of all tested compounds and their molecular weight.

Compound ID	Core Structure	R^1^	R^2^	MW
**1**	Quinazoline	–phenyl	–H	326.4
**2**		–phenyl	–4-morpholino	411.5
**3**		–phenyl	–4-SO_2_N(CH_3_)_2_	433.5
**4**		–phenyl	–4-SO_2_NH_2_	405.5
**5**		–phenyl	–3-SO_2_NH_2_	405.5
**6**		–2-thiophene	–H	332.4
**7**		–2-thiophene	–4-NHCONHPh	466.6
**8**		–2-thiophene	–4-morpholino	417.5
**9**		–2-thiophene	–4-SO_2_NH_2_	411.5
**10**		–2-thiophene	–4-CONH_2_	375.5
**11**		–2-thiophene	–3-SO_2_NH_2_	411.5
**12**		–2-thiophene	–3-CONH_2_	375.5
**13**		–furan	–3-SO_2_NH_2_	395.4
**14**	5-chloropyrimidine	–phenyl	–H	310.8

**Table 2:** Solubility, EC_50_, and E_max_ for the vasorelaxant response in both endothelium-intact (E+) and endothelium-denuded (E−) pulmonary arterial rings, % of inhibition to PE-induced contraction, IC_50_ against PDE-5, and A549 cells by quinazoline analogues.

Compound ID	Vaso E+ EC_50_ µM (SEM)	E+ Emax (%)	Vaso E− EC_50_ µM (SEM)	E− Emax (%)	% PE-Contraction Inhibition (SEM)	Sol. pH_7.4_ mg/mL (µM)	PDE-5 IC_50_, µM (S.D.)	A549 IC_50_, µM (S.D.)	SI E+ vs. A549 (PDE-5 vs. A549)
**1**	5.54 * (2.25)	89.98 ± 5.64	13.46 (1.48)	86.41 ± 4.03	-	0.13 (0.41)	1.38 (0.169)	12.29 (1.2)	2.2 (8.9)
**2**	4.98 (2.38)	94.69 ± 3.33	11.96 (3.17)	85.67 ± 6.05	56.62 (5.90)	0.25 (0.60)	0.53 (0.066)	12.1 (3.6)	2.4 (22.8)
**3**	3.59 (1.42)	82.36 ± 5.90	6.25 (1.80)	64.27 ± 5.31	-	0.38 (0.88)	1.26 (0.092)	18.32 (0.82)	5.1 (14.5)
**4**	3.13 * (1.52)	88.48 ± 5.58	8.29 (1.38)	78.53 ± 9.19	-	0.01(0.03)	0.95 (0.035)	22.97 (4.55)	7.3 (24.2)
**5**	1.63 (0.72)	91.68 ± 3.77	1.15 (0.18)	95.83 ± 2.40	-	0.56 (1.37)	0.07 (0.008)	11.15 (1.22)	6.8 (159.3)
**6**	9.42 (2.54)	88.03 ± 7.39	9.51 (1.28)	91.16 ± 5.26	-	0.13 (0.40)	1.44 (0.17)	27.78 (6)	2.9 (19.3)
**7**	4.10 (1.46)	76.03 ± 5.73	>30	22.31 ± 4.40	43.62 (7.02)	0.10 (0.22)	0.78 (0.101)	2.91 (0.73)	0.7 (3.7)
**8**	0.58 *** (0.22)	98.88 ± 0.79	8.50 (1.6)	100	42.43 (5.99)	0.20 (0.48)	0.51 (0.021)	11.37 (3.07)	19.6 (22.3)
**9**	1.03 (0.33)	98.40 ± 1.40	1.72 (0.63)	82.10 ± 8.43	-	0.06 (0.16)	0.22 (0.038)	32.67 (3.61)	31.7 (148.5)
**10**	1.46 (0.43)	90.15 ± 6.48	5.15 (1.57)	90.42 ± 5.30	-	0.45 (1.20)	0.18 (0.016)	28.29 (2.58)	19.4 (157.2)
**11**	2.28 *** (0.74)	98.74 ± 1.26	10.89 (0.51)	87.42 ± 4.66	-	0.14 (0.34)	0.09 (0.011)	15.04 (4.44)	6.6 (167.1)
**12**	4.15 (1.38)	98.69 ± 1.31	8.15 (1.43)	87.11 ± 3.63	-	0.21(0.56)	0.29 (0.098)	15.44 (2.14)	3.7 (53.2)
**13**	6.61 (2.39)	95.46 ± 2.17	3.02 (1.29)	95.61 ± 2.75	45.93 (11.69)	0.15 (0.38)	0.12 (0.023)	26.92 (2.16)	4.1 (224.3)
**14**	4.49 (1.96)	67.80 ± 5.34	10.69 (2.20)	54.74 ± 6.12	-	1.02 (3.27)	>10	>100	>22.3 (>10)
Sildenafil (SIL)	0.14 (0.05)	97.82 ± 2.19	0.89 (0.48)	85.84 ± 6.41	58.33 (1.77)	20.83 (43.88)	0.002 (0.0008)	-	-
Sodium nitro-prusside (SNP)	0.02 (0.01)	99.58 ± 0.42	0.0045 (0.0007)	100	-	-	-	-	-
Prazosin (PZN)	0.03 (0.01)	98.72 ± 1.28	0.093 (0.03)	100	98.01 (1.02)	0.55 (1.42)	-	-	-

Note: * *p* < 0.05, *** *p <* 0.001 E+ EC_50_ vs. E− EC_50._

## Supplementary Materials

The following are available online, Table S1: Substructure searching of the ChEMBL database using the 2,4-diamino quinazoline scaffold identifies a number of different enzyme/receptor targets and cell based phenotypic responses, Table S2: Exemplar Quinazoline structures identified with activities potentially related to vasodilation pathways, Table S3: Table of the experimental PDE-5, vasodilation, cytotoxicity, solubility and other physicochemical properties used to build the PCA model, Table S4: PCA model coefficients for multivariate analysis of experimental PDE-5 inhibition, vasodilation, cytotoxicity, solubility and other physicochemical properties.

## References

[1] Humbert M., Sitbon O., Chaouat A., Bertocchi M., Habib G., Gressin V., Yaici A., Weitzenblum E., Cordier J.-F., Chabot F. (2006). Pulmonary arterial hypertension in France: Results from a national registry. Am. J. Respir. Crit. Care Med..

[2] Santos-Ribeiro D., Mendes-Ferreira P., Maia-Rocha C., Adao R., Leite-Moreira A.F., Brás-Silva C. (2016). Pulmonary arterial hypertension: Basic knowledge for clinicians. Arch. Cardiovasc. Dis..

[3] Hoeper M.M., Gibbs J.S.R. (2014). The changing landscape of pulmonary arterial hypertension and implications for patient care. Eur. Respir. Rev..

[4] Giaid A., Saleh D. (1995). Reduced expression of endothelial nitric oxide synthase in the lungs of patients with pulmonary hypertension. N. Engl. J. Med..

[5] Christman B.W., McPherson C.D., Newman J.H., King G.A., Bernard G.R., Groves B.M., Loyd J.E. (1992). An imbalance between the excretion of thromboxane and prostacyclin metabolites in pulmonary hypertension. N. Engl. J. Med..

[6] Liu R., Zhang Q., Luo Q., Qiao H., Wang P., Yu J., Cao Y., Lu B., Qu L. (2017). Norepinephrine stimulation of alpha1D-adrenoceptor promotes proliferation of pulmonary artery smooth muscle cells via ERK-1/2 signaling. Int. J. Biochem. Cell Biol..

[7] Sahara M., Takahashi T., Imai Y., Nakajima T., Yao A., Morita T., Hirata Y., Nagai R. (2006). New insights in the treatment strategy for pulmonary arterial hypertension. Cardiovasc. Drugs Ther..

[8] Sitbon O., Humbert M., Jagot J., Taravella O., Fartoukh M., Parent F., Herve P., Simonneau G. (1998). Inhaled nitric oxide as a screening agent for safely identifying responders to oral calcium-channel blockers in primary pulmonary hypertension. Eur. Respir. J..

[9] Badlam J.B., Bull T.M. (2017). Steps forward in the treatment of pulmonary arterial hypertension: Latest developments and clinical opportunities. Ther. Adv. Chronic Dis..

[10] McLaughlin V., Sitbon O., Badesch D., Barst R., Black C., Galie N., Rainisio M., Simonneau G., Rubin L. (2005). Survival with first-line bosentan in patients with primary pulmonary hypertension. Eur. Respir. J..

[11] Simonneau G., Rubin L.J., Galie N., Barst R.J., Fleming T.R., Frost A.E., Engel P.J., Kramer M.R., Burgess G., Collings L. (2008). Addition of sildenafil to long-term intravenous epoprostenol therapy in patients with pulmonary arterial hypertension: A randomized trial. Ann. Intern. Med..

[12] Michelakis E.D., Tymchak W., Noga M., Webster L., Wu X.-C., Lien D., Wang S.-H., Modry D., Archer S.L. (2003). Long-term treatment with oral sildenafil is safe and improves functional capacity and hemodynamics in patients with pulmonary arterial hypertension. Circulation.

[13] Sebkhi A., Strange J.W., Phillips S.C., Wharton J., Wilkins M.R. (2003). Phosphodiesterase type 5 as a target for the treatment of hypoxia-induced pulmonary hypertension. Circulation.

[14] Nagendran J., Archer S.L., Soliman D., Gurtu V., Haromy A., Rebeyka I.M., Ross D.B., Light P.E., Michelakis E.D. (2007). Phosphodiesterase-5 is highly expressed in the hypertrophied human right ventricle and acute inhibition causes increased contractility via cGMP-inhibition of phosphodiesterase-3. Circulation.

[15] Corbin J.D., Francis S.H. (1999). Cyclic GMP phosphodiesterase-5: Target of sildenafil. J. Biol. Chem..

[16] Czarniecki M., Ahn H.-S., Sybertz E.J. (1996). Inhibitors of types I and V phosphodiesterase: Elevation of cGMP as a therapeutic strategy. Ann. Rep. Med. Chem..

[17] Raiesdana A., Loscalzo J. (2006). Pulmonary arterial hypertension. Ann. Med..

[18] Beavo J.A. (1995). Cyclic nucleotide phosphodiesterases: Functional implications of multiple isoforms. Physiol. Rev..

[19] Ückert S., Hedlund P., Andersson K.-E., Truss M.C., Jonas U., Stief C.G. (2006). Update on phosphodiesterase (PDE) isoenzymes as pharmacologic targets in urology: Present and future. Eur. Urol..

[20] Rotella D.P. (2001). Phosphodiesterase type 5 inhibitors: Discovery and therapeutic utility. Drugs Future.

[21] Wang D., Gao F. (2013). Quinazoline derivatives: Synthesis and bioactivities. Chem. Cent. J..

[22] Verhaeghe P., Dumètre A., Castera-Ducros C., Hutter S., Laget M., Fersing C., Prieri M., Yzombard J., Sifredi F., Rault S. (2011). 4-Thiophenoxy-2-trichloromethyquinazolines display in vitro selective antiplasmodial activity against the human malaria parasite Plasmodium falciparum. Bioorg. Med. Chem. Lett..

[23] Chiou W.-F., Liao J.-F., Shum A.Y.-C., Chen C.-F. (1996). Mechanisms of vasorelaxant effect of dehydroevodiamine: A bioactive isoquinazolinocarboline alkaloid of plant origin. J. Cardiovas. Pharmacol..

[24] Abou-Seri S.M., Abouzid K., El Ella D.A.A. (2011). Molecular modeling study and synthesis of quinazolinone-arylpiperazine derivatives as α_1_-adrenoreceptor antagonists. Eur. J. Med. Chem..

[25] Kim Y.H., Choi H., Lee J., Hwang I.-C., Moon S.K., Kim S.J., Lee H.W., Lee S.S., Ahn S.K., Kim S.W. (2008). Quinazolines as potent and highly selective PDE5 inhibitors as potential therapeutics for male erectile dysfunction. Bioorg. Med. Chem. Lett..

[26] Pobsuk N., Urooj Paracha T., Chaichamnong N., Salaloya N., Suphakun P., Hannongbua S., Choowongkomon K., Pekthong P., Chootip C., Ingkaninan K. Design, synthesis and evaluation of *N*^2^,*N*^4^-diaminoquinazoline-based inhibitors of phosphodiesterase type 5. Bioorg. Med. Chem. Lett..

[27] Toviwek B., Suphakun P., Choowongkomon K., Hannongbua S., Gleeson M.P. (2017). Synthesis and evaluation of the NSCLC anti-cancer activity and physical properties of 4-aryl-*N*-phenylpyrimidin-2-amines. Bioorg. Med. Chem. Lett..

[28] Kaneda T., Sasaki N., Urakawa N., Shimizu K. (2016). Endothelium-dependent and-independent vasodilator effects of dimethyl sulfoxide in rat aorta. Pharmacology.

[29] ChEMBL. https://www.ebi.ac.uk/chembl/.

[30] Cai Z., Lu Q., Ding Y., Wang Q., Xiao L., Song P., Zou M.-H. (2015). Endothelial nitric oxide synthase-derived nitric oxide prevents dihydrofolate reductase degradation via promoting S-nitrosylation. Arterioscler. Thromb. Vasc. Biol..

[31] Capettini L.S.A., Cortes S.F., Lemos V.S. (2010). Relative contribution of eNOS and nNOS to endothelium-dependent vasodilation in the mouse aorta. Eur. J. Pharmacol..

[32] El-Daly M., Pulakazhi Venu V.K., Saifeddine M., Mihara K., Kang S., Fedak P.W.M., Alston L.A., Hirota S.A., Ding H., Triggle C.R. (2018). Hyperglycaemic impairment of PAR2-mediated vasodilation: Prevention by inhibition of aortic endothelial sodium-glucose-co-transporter-2 and minimizing oxidative stress. Vascul. Pharmacol..

[33] Lin S., Page N.A., Fung S.M., Fung H.-L. (2013). In vitro organic nitrate bioactivation to nitric oxide by recombinant aldehyde dehydrogenase 3A1. Nitric Oxide.

[34] Kruangtip O., Chootip K., Temkitthawon P., Changwichit K., Chuprajob T., Changtam C., Suksamrarn A., Khorana N., Scholfield C.N., Ingkaninan K. (2015). Curcumin analogues inhibit phosphodiesterase-5 and dilate rat pulmonary arteries. J. Pharm. Pharmacol..

[35] Wisutthathum S., Demougeot C., Totoson P., Adthapanyawanich K., Ingkaninan K., Temkitthawon P., Chootip K. (2018). Eulophia macrobulbon extract relaxes rat isolated pulmonary artery and protects against monocrotaline-induced pulmonary arterial hypertension. Phytomedicine.

[36] Moohammadaree A., Changtam C., Wicha P., Suksamrarn A., Tocharus J., Tocharus C. (2015). Mechanisms of vasorelaxation induced by hexahydrocurcuminin isolated rat thoracic aorta. Phytother. Res..

[37] Pantan R., Onsa-ard A., Tocharus J., Wonganan O., Suksamrarn A., Tocharus C. (2014). Endothelium-independent vasorelaxation effects of 16-*O*-acetyldihydroisosteviol on isolated rat thoracic aorta. Life Sci..

[38] Phuangsawai O., Beswick P., Ratanabunyong S., Tabtimmai L., Suphakun P., Obounchoey P., Srisook P., Horata N., Chuckowree I., Hannongbua S. (2016). Evaluation of the anti-malarial activity and cytotoxicity of 2, 4-diamino-pyrimidine-based kinase inhibitors. Eur. J. Med. Chem..

[39] ChemAxon JChem. www.chemaxon.com.

[40] Umetrics. www.umetrics.com.

