# Supplementary material for: Elucidation of Vasodilation Response and Structure Activity Relationships of N2,N4-Disubstituted Quinazoline 2,4-Diamines in a Rat Pulmonary Artery Model
Source: Molecules. 2019 Jan 14;24(2):281. doi: 10.3390/molecules24020281 (PMC6358775; doi:10.3390/molecules24020281)
Supplement: Supplementary file 1 [file molecules-24-00281-s001.pdf]

Supplementary Data

Elucidation of Vasodilation Response and Structure Activity Relationships of *N*[2],*N*[4]-Disubstituted Quinazoline 2,4-Diamines in a Rat Pulmonary Artery Model

Tamkeen Urooj Paracha [1], Nattakarn Pobsuk [2], Nattapas Salaloy [2], Praphasri Suphakun [3], Dumrongsak Pekthong [1], Supa Hannongbua [2], Kiattawee Choowongkomon [3], Nantaka Khorana [4], Prapapan Temkitthawon [5], Kornkanok Ingkaninan [5], M. Paul Gleeson [6,*] and Krongkarn Chootip [7,*]

[1] Department of Pharmacy Practice, Faculty of Pharmaceutical Sciences, Naresuan University, Phitsanulok 65000, Thailand; ctamz@hotmail.com (T.U.P.); dumrongsakp@yahoo.com (D.P.)

[2] Department of Chemistry, Faculty of Science, Kasetsart University, Bangkok 10900, Thailand; sai-nurse@hotmail.com (N.P.); arthahaha@hotmail.com (N.S.); fscisph@ku.ac.th (S.H.)

[3] Department of Biochemistry, Faculty of Science, Kasetsart University, Bangkok 10900, Thailand; cup14t@gmail.com (P.S.); fsciktc@ku.ac.th (K.C.)

[4] Division of Pharmaceutical Sciences, School of Pharmaceutical Sciences, University of Phayao, Phayao 56000, Thailand; nantaka@hotmail.com

[5] Department of Pharmaceutical Chemistry and Pharmacognosy, Faculty of Pharmaceutical Sciences and Center of Excellence for Innovation in Chemistry, Naresuan University, Phitsanulok 65000, Thailand; prapapantem@gmail.com (P.T.); kornkanoki@nu.ac.th (K.I.)

[6] Department of Biomedical Engineering, Faculty of Engineering, King Mongkut's Institute of Technology Ladkrabang, Bangkok 10520, Thailand; paul.gl@kmitl.ac.th

[7] Department of Physiology, Faculty of Medical Science, Naresuan University, Phitsanulok 65000, Thailand; krongkarnc@nu.ac.th

* Correspondence: krongkarnc@nu.ac.th (K.C.); paul.gl@kmitl.ac.th (M.P.G.); Tel.: +66-55-964658 (K.C.); +66-869779678 (M.P.G.)

Table S1 Substructure searching of the ChEMBL database using the 2,4 diamino quinazoline scaffold identifies a number of different enzyme/receptor targets and cell based phenotypic responses. 2

Table S2 Exemplar Quinazoline structures identified with activities potentially related to vasodilation pathways. 3

Table S3 Table of the experimental PDE-5 inhibition, vasodilation, cytotoxicity, solubility and other physicochemical properties used to build the PCA model. 4

Table S4 PCA model coefficients for multivariate analysis of experimental PDE-5 inhibition, vasodilation, cytotoxicity, solubility and other physicochemical properties. 5

Table S1 Substructure searching of the ChEMBL database using the 2,4 diamino quinazoline scaffold identifies a number of different enzyme/receptor targets and cell based phenotypic responses.

Target/Cell Description	count	Target	Comment
Total (quinazoline activities)	6574	-	
Dihydrofolate reductase	*977*	*Protein/Receptor*	*Potential for pathway modulation [1]*
TCF4/beta-catenin	487	Protein/Receptor	No direct link
Plasmodium falciparum	381	Cell	N/A
Adrenergic receptor alpha-1	222	Protein/Receptor	Implicated in Vasodilation
Alpha-1a adrenergic receptor	197	Protein/Receptor	Implicated in Vasodilation
Alpha-1b adrenergic receptor	158	Protein/Receptor	Implicated in Vasodilation
Histamine H4 receptor	147	Protein/Receptor	No direct link
Alpha-1d adrenergic receptor	136	Protein/Receptor	Implicated in Vasodilation
Histone-lysine N-methyltransferase, H3 lysine-9 specific 3	117	Protein/Receptor	No direct link
Adrenergic receptor alpha-2	106	Protein/Receptor	Implicated in Vasodilation
HT-29	98	Cell	N/A
HCT-116	97	Cell	N/A
Glucose transporter	*83*	*Protein/Receptor*	*Potential for pathway modulation [2]*
C-C chemokine receptor type 4	79	Protein/Receptor	N/A
Leishmania donovani	66	Cell	N/A
Microtubule-associated protein tau	63	Cell	N/A
Neuropeptide Y receptor type 5	62	Protein/Receptor	
PC-3	55	Cell	N/A
Potassium-transporting ATPase	55	Protein/Receptor	No direct link
Nuclear receptor ROR-gamma	51	Protein/Receptor	No direct link
Alpha-2a adrenergic receptor	50	Protein/Receptor	Implicated in Vasodilation
Lysine-specific demethylase 4D-like	50	Protein/Receptor	No direct link
SW480	49	Cell	N/A
Aldehyde dehydrogenase 1A1	*46*	*Protein/Receptor*	*Potential for pathway modulation [3]*
Lysosomal alpha-glucosidase	46	Protein/Receptor	No direct link
Endoplasmic reticulum-associated amyloid beta-peptide-binding protein	41	Protein/Receptor	No direct link
Hexose transporter 1	41	Protein/Receptor	No direct link
Beta amyloid A4 protein	40	Protein/Receptor	No direct link
Melanin-concentrating hormone receptor 1	40	Protein/Receptor	No direct link
N-lysine methyltransferase SETD8	38	Protein/Receptor	No direct link
MDA-MB-231	37	Cell	N/A
4'-phosphopantetheinyl transferase	33	Protein/Receptor	No direct link
Cellular tumor antigen p53	33	Protein/Receptor	No direct link
DU-145	33	Cell	N/A
15-hydroxyprostaglandin dehydrogenase	32	Protein/Receptor	
HepG2	31	Cell	N/A

Table S2 Exemplar Quinazoline structures identified with activities potentially related to vasodilation pathways.

Name	Structure	Status	Activity (nM)	Target
Prazosin	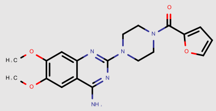	Marketed Drug	1	α1 adrenergic receptor antagonist
Alfuzosin	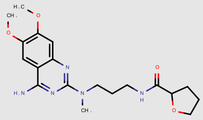	Marketed Drug	3	α1 adrenergic receptor antagonist
Terazosin	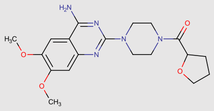	Marketed Drug	7	α1 adrenergic receptor antagonist
Trimetrexate	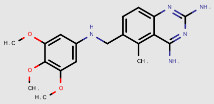	Marketed Drug	3	DHFR***[1]***
CHEMBL1818128	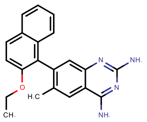	R&D Screening	4.5	DHFR***[1]***
CHEMBL582356	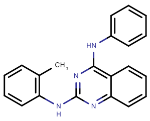	R&D Screening	4496	Glucose Transporter***[2]***
CHEMBL527902	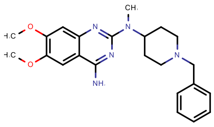	R&D Screening	4087	Glucose Transporter***[2]***
CHEMBL546792	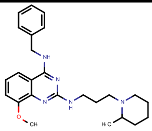	R&D Screening	4102	Glucose Transporte r***[2]***
PRAZOSIN HYDROCHLORIDE	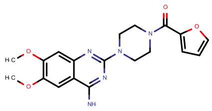	R&D Screening	25000	Aldehyde dehydrogenase 1A1***[3]***

Table S3 Table of the experimental PDE-5, vasodilation, cytotoxicity, solubility and other physicochemical properties used to build the PCA model.

ID	Log Sol	PDE-5 pIC_{50}	E+ Vaso. pIC_{50}	E- Vaso. pIC_{50}	Cytox. pIC_{50}	JCHEM MOST_APKA	JCHEM MOST_BPKA	JLogP	JLogD	JCMass	Chiral	Rings	Aring	PSA	ROT	Heavy Atoms	ALI	AROM	AROM/ ALI RATIO	HBA	HBD
1	-0.39	5.86	5.26	4.87	4.91	13.69	4.93	5.34	5.33	326.40	0	4	4	49.84	5	25	3	22	7.33	4	2
6	-0.40	5.84	5.03	5.03	4.56	13.69	4.93	5.25	5.25	332.43	0	4	4	49.84	5	24	3	21	7.00	4	2
11	-0.47	7.05	5.64	4.96	4.82	10.24	4.64	3.85	3.85	411.50	0	4	4	110.00	6	28	7	21	3.00	6	3
8	-0.32	6.29	6.24	5.07	4.94	15.02	5.30	5.14	5.13	417.53	0	5	4	62.31	6	30	9	21	2.33	6	2
12	-0.25	6.54	5.38	5.09	4.81	13.03	4.75	4.10	4.10	375.45	0	4	4	92.93	6	27	6	21	3.50	5	3
10	0.08	6.75	5.84	5.29	4.55	12.90	4.70	4.10	4.10	375.45	0	4	4	92.93	6	27	6	21	3.50	5	3
9	-0.80	6.65	5.99	5.76	4.49	10.69	4.54	3.85	3.85	411.50	0	4	4	110.00	6	28	7	21	3.00	6	3
4	-1.52	6.02	5.50	5.08	4.64	10.69	4.55	3.94	3.94	405.48	0	4	4	110.00	6	29	7	22	3.14	6	3
2	-0.22	6.28	5.30	4.92	4.92	15.02	5.31	5.23	5.22	411.51	0	5	4	62.31	6	31	9	22	2.44	6	2
7	-0.66	6.11	5.39	4.52	5.54	11.75	4.90	6.39	6.39	466.56	0	5	5	90.97	7	34	7	27	3.86	5	4
14	0.51	5.00	5.35	4.97	4.00	13.54	3.57	4.56	4.56	310.79	0	3	3	49.84	5	22	4	18	4.50	4	2
13	-0.42	6.94	5.18	5.52	4.57	10.24	4.62	3.00	3.00	395.44	0	4	4	123.14	6	28	7	21	3.00	6	3
3	-0.06	5.90	5.45	5.20	4.74	12.28	4.54	4.39	4.39	433.53	0	4	4	87.22	6	31	9	22	2.44	6	2

ID	Log Sol	PDE-5 pIC50	E+ Vaso pIC50	E- Vasop IC50	Cytox. pIC50	JCHEM MOST_APKA	JCHEM MOST_BPKA	JLogP	JLogD	JCMass	Chiral	Rings	Aring	PSA	ROT	Heavy Atoms	ALI	AROM	AROM/ALI RATIO	HBA	HBD
5	0.14	7.14	5.79	5.94	4.95	10.24	4.64	3.94	3.94	405.48	0	4	4	110.00	6	29	7	22	3.14	6	3

Table S4 PCA model coefficients for multivariate analysis of experimental PDE-5 inhibition, vasodilation, cytotoxicity, solubility and other physicochemical properties.

Component	R2X	R2X(cum)	Eigenvalue	Q2	Limit	Q2(cum)	Significance	Iterations
1	0.415	0.415	5.8	0.0799	0.118	0.0799	R2	25
2	0.284	0.699	3.98	0.257	0.126	0.316	R1	10
3	0.116	0.815	1.63	0.12	0.134	0.398	R2	17
4	0.0623	0.877	0.872	-0.0663	0.144	0.358	R2	46

References

[1] Crabtree, M.J.; Hale, A.B.; Channon, K.M., Dihydrofolate reductase protects endothelial nitric oxide synthase from uncoupling in tetrahydrobiopterin deficiency. *Free Radical Biology and Medicine,* **2011**, *50*, (11), 1639-1646.

[2] El-Daly, M.; Pulakazhi Venu, V.K.; Saifeddine, M.; Mihara, K.; Kang, S.; Fedak, P.W.M.; Alston, L.A.; Hirota, S.A.; Ding, H.; Triggle, C.R.; Hollenberg, M.D., Hyperglycaemic impairment of PAR2-mediated vasodilation: Prevention by inhibition of aortic endothelial sodium-glucose-co-Transporter-2 and minimizing oxidative stress. *Vascular Pharmacology,* **2018**, *109*, 56-71.

[3] Lin, S.; Page, N.A.; Fung, S.M.; Fung, H.-L., In vitro organic nitrate bioactivation to nitric oxide by recombinant aldehyde dehydrogenase 3A1. *Nitric Oxide,* **2013**, *35*, 137-143.
